# Evaluating the Duke Criteria for infectious endocarditis in a single-center retrospective study

**DOI:** 10.1038/s41598-024-70196-x

**Published:** 2024-08-22

**Authors:** Sascha d’Almeida, Kathrin Reischmann, Stefanie Andreß, Dominik Felbel, Tilman Stephan, Birgit Hay, Friederike Rohlmann, Dominik Buckert, Wolfgang Rottbauer, Sinisa Markovic

**Affiliations:** 1 Department of Medicine II, Ulm University Medical Center, Ulm, Germany; 2 Department of Anesthesiology and Intensive Care, Ulm University Medical Center, Ulm, Germany; 3 Department of Medical Biometry and Statistics, Ulm University Medical Center, Ulm, Germany

**Keywords:** Infective endocarditis, Duke criteria, Major criterion, Minor criterion, Cardiac imaging, Vegetations, Microhematuria, Prosthetic heart valve, Rheumatic disease, Cardiology, Valvular disease

## Abstract

The Duke Criteria have shaped the way infectious endocarditis (IE) is diagnosed in the last 30 years. This study aims to evaluate their current validity and importance in the diagnostic of IE. A retrospective cohort study was conducted on 163 consecutive patients who presented at the University Hospital in Ulm (Germany) with clinical suspicion of IE between 2009 and 2019. With patients’ medical records we differentiated between definitive endocarditis (DIE), possible endocarditis (PIE) and rejected endocarditis (RIE) and assessed the validity of the Duke Criteria in comparison to the final discharge diagnosis. We then tried to identify new potential parameters as an addition to the current valid Duke Criteria. The validity of the Duke Criteria improves with the length of hospitalization (especially cardiac imaging criterion, RIE 33.3%, PIE 31.6% and DIE 41.9%, *p* = 0.622 at admission and RIE 53.3%, PIE 68.4%, DIE 92.2%, *p* < 0.001 at discharge). At admission, overall sensitivity and specificity were respectively 29.5 and 91.2% in the DIE group. At discharge, sensitivity in the DIE group rose to 77.5% and specificity decreased to 79.4%. Of all screened metrics, microhematuria (*p* = 0.124), leukocyturia, (*p* = 0.075), younger age (*p* = 0.042) and the lack of rheumatoid disease (*p* = 0.011) showed a difference in incidence (*p* < 0.2) when comparing DIE and RIE group. In multivariate regression only microhematuria qualified as a potential sixth minor criterion at admission. Even with the latest technological breakthroughs our findings suggest that the Duke Criteria continue to hold value in the accurate assessment of IE. Future efforts must shorten the time until diagnosis.

## Introduction

Since the latest change of the ESC guidelines on antibiotic prophylaxis in infectious endocarditis^1^ and the increase of surgical and interventional cardiac procedures like ICDs, pacemakers or TAVI, the nature of infectious endocarditis has changed a lot. The incidence of endocarditis has been increasing^2^ and the mean age of patients with endocarditis has risen^3,4^ from around 52–57 years in the late 90s to 63 years in the latest study in 2022^5^. The bacterial spectrum that leads to IE has also evolved^6^. Therefore, diagnosis and treatment of IE have become more complex. According to the European Register of Endocarditis^7^ between 2016 and 2018, 56.6% of infective endocarditis affected a native valve (NVE), 30.1% a prosthetic valve (PVE) and 9.9% an intracardiac device (CIED) such as a pacemaker or ICD lead^8^.

Even though lab methods, surgical safety and cardiac imaging have improved a lot, mortality did not improve in these cohorts^9^.

The Duke Criteria that were first introduced in 1994 have widely been used in the past to assess the likelihood of IE in patients with suspected valvular or vascular involvement^10^. However, the validity of these criteria in the current clinical setting remains uncertain because of said paradigm shift with increasing prosthetic valve and lead endocarditis^7,11–13^.

Since the establishing of the Duke Criteria, there have been constant efforts to improve or modify them. A British study of 1997 tried to expand the minor criteria and increased the diagnosis rate of definitive endocarditis by 10 points in native IE and 39 points in prosthetic IE in comparison to the unmodified Duke Criteria^4^. Among the new minor criteria that were suggested, there were petechiae, an elevated high erythrocyte sedimentation rate, a high C-reactive protein (CRP) level; a peripheral or central nonfeeding catheter, and microhematuria^4^.

## Methods

In order to assess the timeliness of the Duke Criteria, we retrospectively analyzed a cohort of 163 consecutive patients that presented with suspected endocarditis. Because of the heterogenous presentation of IE, we followed the ESC recommendations to classify the patients into three groups: we differentiated between a definitive endocarditis (DIE), a possible endocarditis (PIE) and a rejected endocarditis (RIE). We used the definitive discharge diagnosis as a gold standard and applied the Duke Criteria at different moments, within the first 24 h and at discharge to assess their validity. We gathered lab values, clinical data, imaging data and anamnestic data in order to find new parameters that correlate with the incidence of IE.

In the statistical analysis of nominal variables the Fisher's-Exact-Test was used for 2 × 2 tables and for 3 × 2 tables with expected small numbers the extended Exact Fisher-Freeman-Halton Test was used to provide more exact results. For the comparison of 3 groups and 3 or more variables and the Chi-Square-Test was used. For the comparison of metric variables, the Mann–Whitney-U-Test was used for 2 groups and the Kruskal–Wallis-Test for 3 groups. Significance level was *p* < 0.05.

In order to find new potential variables to improve the existing Duke Criteria, we primary did a screening for any differences of incidence in the DIE and RIE group. All parameters with a *p* value < 0.2 in this screening (tested via Fisher’s-Exact-Test, Exact Fisher-Freeman-Halton Test, Chi-Square-Test, Kruskal–Wallis-Test and Mann–Whitney-U-Test) as well as the existing Duke Criteria were further analyzed in regression models.

First, we analyzed the existing Duke Criteria and the potential new parameters in a univariate binary logistic regression for the DIE and RIE group with calculation of the Odds Ratio and their 95% confidence interval. Irrespective the *p* values in this univariate regression, the Duke Criteria as well as the potential new parameters were put into a multivariate binary logistic regression model with forward selection within the DIE and RIE group. Significance level of the logistic regressions was *p* < 0.05.

### Ethical approval

The methods employed in this manuscript were meticulously executed in strict accordance with pertinent guidelines, regulations, and ethical standards outlined by the Federal Republic of Germany. We assure the ethical soundness of the conducted research and do not violate any federal or national law. All experimental protocols detailed in this manuscript were thoroughly reviewed and approved by the Institute of epidemiology and the Institute of History Theory and Ethics in Medicine in Ulm.

### Informed consent

I confirm that informed consent was diligently obtained from all subjects involved in the study, as well as from their legal guardian(s) where applicable. This critical ethical protocol ensures the rights, welfare, and confidentiality of all participants throughout the research process.

## Results

### Baseline characteristics and study population

**Figure 1:**
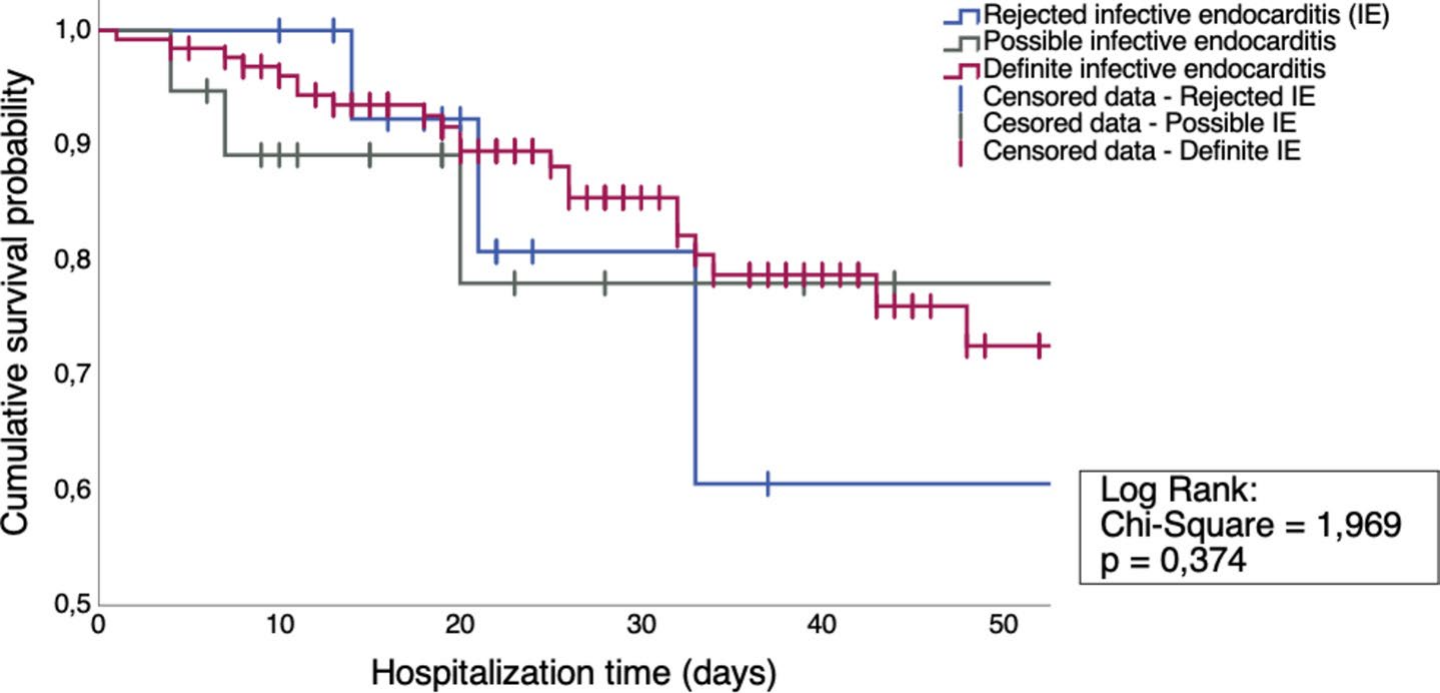
Kaplan Maier Survival Analysis of all IE groups within the first 50 days of hospitalization.

Within our cohort of 163 patients, 118 (72.4%) were male patients. The median age was 68.6 years (IQR 58.0–77.6). DIE group patients were significantly younger (*p* = 0.007). Infectious endocarditis was rejected (RIE) in 15 patients, 19 cases were discharged as a possible endocarditis (PIE) and in 129 patients IE was confirmed and therefore definitive (DIE) (Fig. 1). The differential diagnosis in the RIE group were mostly sepsis or from the rheumatoid disease spectrum. Within the 129 patients, 14 patients (10.9%) had more than one infected valve. The most common infected valve was the aortic valve with 59 cases (41.3%), followed by the mitral valve in 57 patients (39.9%). Lead infections occurred in 15 cases (10.5%). In 11 (7.7%) patients the tricuspid valve was infested and 1 patient had an endocarditis of the pulmonary valve. PVE occurred in 33 cases and NVE in 84 patients.

The median hospitalization length was 23 days, of which roughly half of the time (11 days) was spent on an intensive care unit. Half of our cohort (48.5%) had to go to an intensive care unit. During the clinical stay, overall mortality was 18.4% (Table 1 and Fig. 1). Within these 18.4% of patients, the median time of death was 20 days after admission (IQR 10.8–33.0). Patients who had a rejected endocarditis showed a non-significantly higher mortality rate within the clinical stay (26.7%) than the other groups (17.1%), *p* = 0.481). The median time that elapses until IE diagnosis was 2 days. The median time until IE rejection was 8 days.

**Table 1:** Patient characteristics for all patients and the patient groups “Rejected IE”, “Possible IE” and “Definite IE”.

Patient characteristics	Overall (n = 163)	Rejected IE (n = 15)	Possible IE (n = 19)	Definite IE (n = 129)	*p* value^1^
Gender					
Male, n (%)	118 (72.4)	12 (80)	10 (52.6)	96 (74.4)	*0.121*
Age (years)					
Total, median (IQR)	68.6 (58–77.6)	73.7 (65.8–80.7)	75.1 (67.7–88.1)	65.9 (55.2–75.6)	***0.007***
Prosthetic valve before presentation					
Total, n (%)	46 (28.2)	5 (33.3)	4 (21.1)	37 (28.7)	*0.7*
CIED before presentation					
Total, n (%)	39 (23.9)	5 (33.3)	6 (31.6)	28 (21.7)	*0.42*
Pacemaker, n (%)	22 (13.5)	3 (20)	4 (21.1)	15 (11.6)	
Defibrillator, n (%)	8 (4.9)	1 (6.7)	2 (10.5)	5 (3.9)	*0.687*
CRT-D, n (%)	9 (5.5)	1 (6.7)	0 (0)	8 (6.2)	
Diagnostic delay after presentation					
Days elapsed until final diagnosis, median (IQR)	4 (2–9)	13 (4.8–25.3)	4 (2–10)	3 (2–7)	***0.010***
Hospitalization time, Median (IQR)	23 (16–39)	21 (16–33)	19 (10–39)	25 (18–40)	*0.208*
Intensive care treatment					
Patient with intensive care treatment, n (%)	79 (48.5)	5 (33.3)	7 (36.8)	67 (51.9)	*0.261*
Length of the stay (days), median (IQR)	11 (5–17)	19 (8.5–36)	6 (2–19)	10 (5–16)	*0.37*
In-hospital mortality					
Total, n (%)	30 (18.4)	4 (26.7)	4 (21.1)	22 (17.1)	*0.58*
Day of death after admission^2^, median (IQR)	20 (10.75–33)	27 (15.8–72.8)	13.5 (4.8–47.8)	20 (10.8–32.3)	

*P* value^1^ refers to the comparison between the groups “Rejected IE”, “Suspected IE”, Definite IE”. For nominal variables the Exact Fisher-Freeman-Halton-Test respectively the Chi-Square-Test was used and for metric variables the Kruskal–Wallis-Test was used.

^2^The *p* value of 0.007 refers to the age of the different groups.

In hospital mortality only counts patients that died during the stay in our hospital. Survival is compared via log rank test in Fig. 1.

*IE* infective endocarditis, *IQR* interquartile range, *n* number of patients, *CIED* cardiac implantable electronic device, *CRT-D* cardiac resynchronization therapy.

Significant values are in bold italic.

### ESC 2015 Duke Criteria

Table 2 shows all Duke Criteria at the day of admission and until discharge for our collective. At admission within the major Duke Criteria, cardiac imaging did not show significantly more vegetations or IE specific degenerations between the three groups (RIE 33.3%, PIE 31.6% and DIE 41.9%, *p* = 0.622) while microbiology was significantly more often positive (RIE 40.0%, PIE 10.5% DIE 45%, *p* = 0.015). Upon discharge the significance of the cardiac imaging criterion improved greatly (RIE 53.3%, PIE 68.4%, DIE 92.2%, *p* < 0.001) as well as the significance of the microbiologic criterion (RIE 40.0%, PIE 36.8% DIE 69.8%, *p* = 0.003). At admission none of the minor criteria were significant. Upon discharge, vascular phenomena were the only criterion that occurred significantly more often in the DIE group (RIE 13.3%, PIE 21.1% and DIE 52.7%, *p* < 0.001).

**Table 2:** ESC 2015 Duke-Criteria on the day of admission and until discharge for the patient groups “Rejected IE”, “Possible IE” and “Definite IE”.

	Positive ESC 2015 Duke-Criteria	Time period	Rejected IE (*n* = 15)	Possible IE (*n* = 19)	Definite IE (*n* = 129)	*p* value^1^
Major criteria	Imaging positive for IE, n (%)	Day of admission	5 (33.3)	6 (31.6)	54 (41.9)	*0.622*
		Until discharge	8 (53.3)	13 (68.4)	119 (92.2)	< ***0.001***
	Blood cultures positive for IE, n (%)	Day of admission	6 (40)	2 (10.5)	58 (45.0)	***0.015***
		Until discharge	6 (40)	7 (36.8)	90 (69.8)	***0.003***
Minor criteria	Fever > 38 °C, n (%)	Day of admission	7 (46.7)	6 (31.6)	66 (51.2)	*0.273*
		Until discharge	8 (53.3)	9 (47.4)	78 (60.5)	*0.551*
	Vascular phenomena, n (%)	Day of admission	2 (13.3)	4 (21.1)	37 (28.7)	*0.427*
		Until discharge	2 (13.3)	4 (21.1)	68 (52.7)	< ***0.001***
	Immunological phenomena, n (%)	Day of admission	1 (6.7)	0 (0)	4 (3.1)	*0.463*
		Until discharge	1 (6.7)	3 (15.8)	15 (11.6)	*0.817*
	Predisposition, n (%)	Day of admission	8 (53.3)	10 (52.6)	78 (60.5)	*0.774*
		Until discharge	8 (53.3)	10 (52.6)	78 (60.5)	*0.774*
	Microbiological evidence, n (%)	Day of admission	0 (0)	0 (0)	11 (8.5)	*0.356*
		Until discharge	0 (0)	1 (5.3)	17 (13.2)	*0.312*

*P* value^1^ refers to the comparison between the groups “Rejected IE”, “Suspected IE”, Definite IE”. For nominal variables the Exact Fisher-Freeman-Halton-Test respectively the Chi-Square-Test was used and for metric variables the Kruskal–Wallis-Test was used.

*IE* infective endocarditis, *n* number of patients.

Significant values are in bold italic.

Within our study population analyzed here, 47.2% of the patients carried implanted foreign material like a prosthetic heart valve or a CIED (Cardiac Implantable Electronic Device) as a predisposition for IE. There was no significant difference in the presence of such a device in the three groups (*p* = 0.700 for prosthetic valves and *p* = 0.420 for CIEDs)**.**

When comparing PVE (n = 33) and NVE (n = 84) in the DIE group, patients with PVE are slightly older (*p* = 0.062) and have more comorbidities like heart failure (*p* = 0.049) (Table 3). Still, most cardiac factors are not significantly different. Most Duke Criteria apply the same in both groups.

**Table 3:** Validity of the ESC 2015 Duke Criteria in NVE (n = 84) and PVE (n = 33) of the DIE group.

Parameter		Native valve endocarditis (*n* = 84)	Prosthetic valve endocarditis (*n* = 33)	*p* value^1^
Gender				
Male, n (%)		60 (71.4)	27 (81.8)	*0.347*
Age (years)				
Total, median (IQR)		65.3 (51.4–73.7)	70 (59.9–80)	*0.062*
Rate of positive Duke-Criteria, n (%)				
Imaging positive for IE	Day of admission	35 (41.7)	13 (39.4)	*1.000*
	Until discharge	77 (91.7)	31 (93.9)	*1.000*
Blood cultures positive for IE	Day of admission	33 (39.3)	17 (51.5)	*0.299*
	Until discharge	56 (66.7)	24 (72.7)	*0.660*
Fever > 38 °C	Day of admission	42 (50)	16 (48.5)	*1.000*
	Until discharge	52 (61.9)	18 (54.5)	*0.532*
Vascular phenomena	Day of admission	23 (27.4)	12 (36.4)	*0.374*
	Until discharge	43 (51.2)	23 (69.7)	*0.097*
Immunological phenomena	Day of admission	3 (3.6)	1 (3)	*1.000*
	Until discharge	8 (9.5)	5 (15.2)	*0.513*
Predisposition	Day of admission	34 (40.5)	33 (100)	** < *0.001***
	Until discharge	34 (40.5)	33 (100)	** < *0.001***
Microbiological evidence	Day of admission	8 (9.5)	2 (6.1)	*0.723*
	Until discharge	12 (14.3)	4 (12.1)	*1.000*
Pre-existing cardiovascular disease, n (%)				
Cardiac arrhythmia		38/84 (45.2)	20/33 (60.6)	*0.154*
CAD		31/84 (36.9)	17/33 (51.5)	*0.210*
Diabetes mellitus		24/84 (28.6)	11/33 (33.3)	*0.657*
Diabetic complications		13/19 (68.4)	2/7 (28.6)	*0.095*
Hypertonia		50/84 (59.5)	23/33 (69.7)	*0.397*
History of HF		34/84 (45.3)	19/28 (67.9)	***0.049***
Open Wounds		9/84 (10.7)	4/33 (12.1)	*1.000*
PAD		7/84 (8.3)	7/33 (21.2)	*0.064*
History of MI		14/84 (16.7)	5/33 (15.2)	*1.000*
Structural heart disease		5/84 (6)	0/33 (0)	*0.320*

*P* value^1^ refers to the p value of the groups “NVE”, “PVE”, compared by Fisher’s Exact Test or Mann–Whitney-U-Test.

*CAD* coronary artery disease, *PAD* peripheral artery disease, *MI* myocardial infarction, *HF* systolic heart failure (Left ventricular ejection fraction < 55%).

Significant values are in bold italic.

When we defined the DIE and PIE group as IE positive (affected) and compared them to IE-free patients, sensitivity and specificity of the Duke Criteria were respectively 56.1 and 40.0% in the DIE and PIE groups together at admission and changed to 94.6 and 33.3% over time.

By only defining the DIE group as IE positive we observed a sensitivity and specificity on the day of admission of respectively 29.5 and 91.2%, which changes to a sensitivity of 77.5% and a specificity of 79.4% on the day of discharge.

### Screening for new potential parameters

Looking for new potential parameters in addition to the current Duke Criteria, we compared multiple variables between all three groups (*p* value 1) and then only between RIE and DIE group (*p* value 2). Only parameters that were significant in both analyses were considered for complementing the Duke Criteria.

Within all new screened metrics that we observed when comparing all 3 groups, age (*p* = 0.007), immunosuppression (*p* = 0.184), rheumatological disease (*p* = 0.012), leukocyturia (*p* = 0.113) until discharge, microhematuria until discharge (*p* = 0.151) creatinine kinase levels (*p* = 0.107), NT-proBNP levels (0.014) and sodium levels (*p* = 0.009) showed a *p* value < 0.2. When comparing these parameters in the DIE and RIE groups, the ones showing a *p* value lower than 0.2 were age (*p* = 0.042), rheumatological disease (*p* = 0.011), leukocyturia, (*p* = 0.075) and microhematuria (*p* = 0.124). They were therefore suspected to possibly have an additional impact on the diagnosis and were put into a uni- and multivariate regression analysis in the next step. Only age was not included because of a big interquartile range overlap, despite the significant difference of the median age of patients in the PIE and DIE group. The IQR of PIE patients was 66–81 years, while the older half of DIE patients had an age of 66–92 years. The overview of all parameters is displayed in Table 4.

**Table 4:** Number of pre-existing diseases, results of urine tests and laboratory parameter on the day of admission for the patient groups “Rejected IE”, “Possible IE” and “Definite IE”.

Parameter		Rejected IE (*n* = 15)	Possible IE (*n* = 19)	Definite IE (*n* = 129)	*p* value^1^	*p* value^2^
Pre-existing cardiovascular disease, n (%)						
Cardiac arrhythmia		8/15 (53.3)	12/19 (63.2)	68/129 (52.7)	*0.716*	
Coronary heart disease		9/15 (60)	8/19 (42.1)	54/129 (41.9)	*0.448*	
Diabetes mellitus		2/15 (13.3)	4/19 (21.1)	39/129 (30.2)	*0.351*	
Diabetic long-term consequences		1/2 (50)	2/4 (50)	19/30 (63.3)	*0.818*	
Hypertension		10/15 (66.7)	15/19 (78.9)	82/129 (63.6)	*0.439*	
Left ventricular ejection fraction < 55%		5/12 (41.7)	9/16 (56.3)	57/113 (50.4)	*0.781*	
Open wounds		4/15 (26.7)	2/19 (10.5)	17/129 (13.2)	*0.349*	
Peripheral artery disease		2/15 (13.3)	3/19 (15.8)	17/129 (13.2)	*0.916*	
Post myocardial infarction		4/15 (26.7)	2/19 (10.5)	21/129 (16.3)	*0.438*	
Structural heart disease		1/15 (6.7)	2/19 (10.5)	9/129 (7)	*0.856*	
Other, n (%)						
Age (years), median (IQR)		73.7 (65.8–80.7)	75.1 (67.7–88.1)	65.9 (55.2–75.6)	***0.007***	***0.042***
Cancer		2/15 (13.3)	1/19 (5.3)	12/129 (9.3)	*0.776*	
Chronic renal insufficiency		6/15 (40)	8/19 (42.1)	37/129 (28.7)	*0.361*	
Dialysis		1/15 (6.7)	0/19 (0)	6/129 (4.7)	*0.609*	
Hypo- or Hyperthyreosis		2/15 (13.3)	4/19 (21.1)	26/129 (20.2)	*0.878*	
Immunosuppression		0/15 (0)	3/19 (15.8)	8/129 (6.2)	***0.184***	*1.000*
Reduced dental status		0/2 (0)	1/2 (50)	9/25 (36)	*0.784*	
Rheumatological disease		4/15 (26.7)	2/19 (10.5)	6/129 (4.7)	***0.012***	***0.011***
Urine test results, n (%)						
Leukocyturia						
On day 1		0/1 (0)	2/7 (28.6)	24/48 (50)	*0.431*	
On day 1–2		1/4 (25)	2/7 (28.6)	28/60 (46.7)	*0.536*	
On day 1–3		1/5 (20)	2/7 (28.6)	29/61 (47.5)	*0.398*	
On day 1–4		1/6 (16.7)	3/8 (37.5)	31/66 (47)	*0.339*	
Until discharge		1/9 (11.1)	4/12 (33.3)	39/87 (44.8)	***0.113***	***0.075***
Microhematuria						
On day 1		0/1 (0)	4/7 (57.1)	35/47 (74.5)	*0.237*	
On day 1–2		2/4 (50)	4/7 (57.1)	45/60 (75)	*0.282*	
On day 1–3		3/5 (60)	4/7 (57.1)	46/61 (75.4)	*0.429*	
On day 1–4		3/6 (50)	5/8 (62.5)	50/66 (75.8)	*0.266*	
Until discharge		4/9 (44.4)	7/12 (58.3)	63/87 (72.4)	***0.151***	***0.124***
Leukocyturia and microhematuria		1/9 (11.1)	4/12 (33.3)	34/87 (39.1)	*0.273*	
Laboratory parameter on the day of admission						
Hemoglobin (g/dl)	n	14/15	19/19	127/129		
	median (IQR)	12.2 (10.6–13.5)	11 (10.5–11.3)	10.8 (9.3–12.7)	*0.259*	
Leukocytes (1000/µl)	n	14/15	19/19	127/129		
	median (IQR)	9.1 (6.5–11)	8.8 (7.1–11.3)	10.3 (7.5–15.2)	*0.205*	
Thrombocytes (1000/µl)	n	14/15	19/19	126/129		
	median (IQR)	193 (126.5–309.5)	148 (97–228)	191.5 (127.8–280.5)	*0.309*	
C-reactive protein (mg/l)	n	14/15	18/19	123/129		
	median (IQR)	49.3 (17.7–155.5)	59.8 (44–83.9)	73.9 (45–147.7)	*0.228*	
Creatine kinase (U/l)	n	10/15	16/19	105/129		
	median (IQR)	77.5 (37.5–145.5)	88 (67.3–145)	52 (34.5–100)	***0.107***	*0.404*
Lactate dehydrogenase (U/l)	n	11/15	17/19	112/129		
	median (IQR)	269 (225–368)	293 (240–458.5)	300.5 (240–392.5)	*0.630*	
NT-proBNP (pg/ml)	n	8/15	9/19	52/129		
	median (IQR)	2292 (867.5–3989.5)	10,800 (7634–35,000)	3878.5 (1112–11,097.8)	***0.014***	*0.344*
Sodium (mmol/l)	n	15/15	19/19	129/129		
	median (IQR)	137 (135–141)	140 (138–142)	137 (133–139.5)	***0.009***	*0.225*
Potassium (mmol/l)	n	14/15	18/19	128/129		
	median (IQR)	3.95 (3.7–4.2)	3.75 (3.6–4.3)	4.0 (3.5–4.5)	*0.769*	
Creatinine (µmol/l)	n	15/15	19/19	129/129		
	median (IQR)	123 (75–185)	132 (73–209)	103 (78.5–162)	*0.772*	

*P* value^1^ refers to the groups “Rejected IE”, “Possible IE” and “Definite IE”. For nominal variables the Exact Fisher-Freeman-Halton-Test respectively the Chi-Square-Test was used and for metric variables the Kruskal–Wallis-Test respectively the Mann–Whitney-U-Test was used.

*P* value^2^ refers to the groups “Rejected IE” and “Definite IE” and was only calculated when the respective *p* value^1^ was < 0.2. Fisher’s-Exact-Test or Mann–Whitney-U-Test was used.

*IE* infective endocarditis, *n* number of patients, *NT-proBNP* N-terminal prohormone of brain natriuretic peptide, *WBC* white blood cell count, *PLT* platelets.

Significant values are in bold italic.

In the following univariate analysis (Table 5) the predictive power of every Duke Criterion and of the three new potential parameters leukocyturia, microhematuria and rheumatological disease was tested. Significant *p* values < 0.005 and an OR > 1 were seen in the Duke Criteria “Cardiac Imaging” (*p* < 0.001), “Positive Microbiology as a major criterion” (*p* = 0.027) and “Vascular phenomena” (*p* = 0.011). The remaining Duke Criteria had no significant predictive power for the probability of IE when positive. Looking at the new potential parameter, a pre-existing rheumatological disease decreased the probability of IE (OR 0.13, *p* = 0.005), leukocyturia and microhematuria had no significant OR.

**Table 5:** Univariate binary logistic regression of the 2015 ESC Duke Criteria and potential new variables for the RIE and DIE groups.

Variable	Odds ratio	95%-CI odds ratio	*p* value
Duke-Criteria (clinical course) (n = 144)			
Cardiac Imaging	10.41	3.13–34.65	** < *0.001***
Major criterion: Positive Microbiology	3.46	1.15–10.39	***0.027***
Fever > 38 °C	1.34	0.46–3.92	*0.595*
Vascular phenomena	7.25	1.57–33.41	***0.011***
Immunologic phenomena	1.84	0.23–15.03	*0.568*
Pre-existing heart disease/Intravenous drug abuse	1.34	0.46–3.92	*0.595*
Minor criterion: Positive Microbiology	–	*–*	*0.998*
New potential parameter			
Leukocyturia (n = 96)	6.500	0.78–54.23	*0.084*
Microhamaturia (n = 96)	3.28	0.81–13.26	*0.095*
Rheumatologic disease (n = 144)	0.13	0.03–0.55	***0.005***

*95%-CI* 95% confidence interval.

Significant values are in bold italic.

Since the Duke Criteria hold value as a score and not as single predictors, all parameters tested in the univariate regression before (Duke Criteria and potential new parameter) were included in a multivariate binary logistic regression model with forward conditional inclusion. Following their ascending *p* values, "Positive Imaging" was introduced in the first step (OR = 59.79, *p* = 0.005) of the regression, followed by the other two significant Duke Criteria "Positive Microbiology" (OR = 14.42, *p* = 0.028) and "Vascular Phenomena" (OR = 21.25, *p* = 0.019). In the fourth step, the new potential variable "Microhematuria” (OR = 12.15, *p* = 0.032) was added to the model. There are no further criteria with significant *p* values, which is why the regression model did not include the remaining Duke Criteria or potential new parameter. The included and non-included variables are presented in Table 6. The regression model is significant in whole (*p* = 0.018, n = 96) and correctly classifies 89 out of 96 patients (92.7%).

**Table 6:** Multivariate binary logistic regression with forward conditional inclusion of the existing Duke Criteria and potential new variables comparing the RIE and DIE groups.

Regression model (n = 96; *p* = 0.018; Validity: 92.7%)			
Included variables	Odds ratio	95%-CI odds ratio	*p* value
Positive imaging	59.79	3.39–1054.36	***0.005***
Positive microbiology (major criterion)	14.42	1.33–156.79	***0.028***
Vascular phenomena	21.25	1.65–273.19	***0.019***
Microhematuria	12.15	1.24–119.17	***0.032***

Excluded variables			*p* value
Fever > 38 °C			*0.918*
Immunologic phenomena			*0.559*
Predisposition			*0.505*
Positive microbiology (minor criterion)			*0.129*
Leukocyturia			*0.117*
Rheumatological disease			*0.104*

*95%-CI* 95% confidence interval.

Significant values are in bold italic.

**Figure 2:**
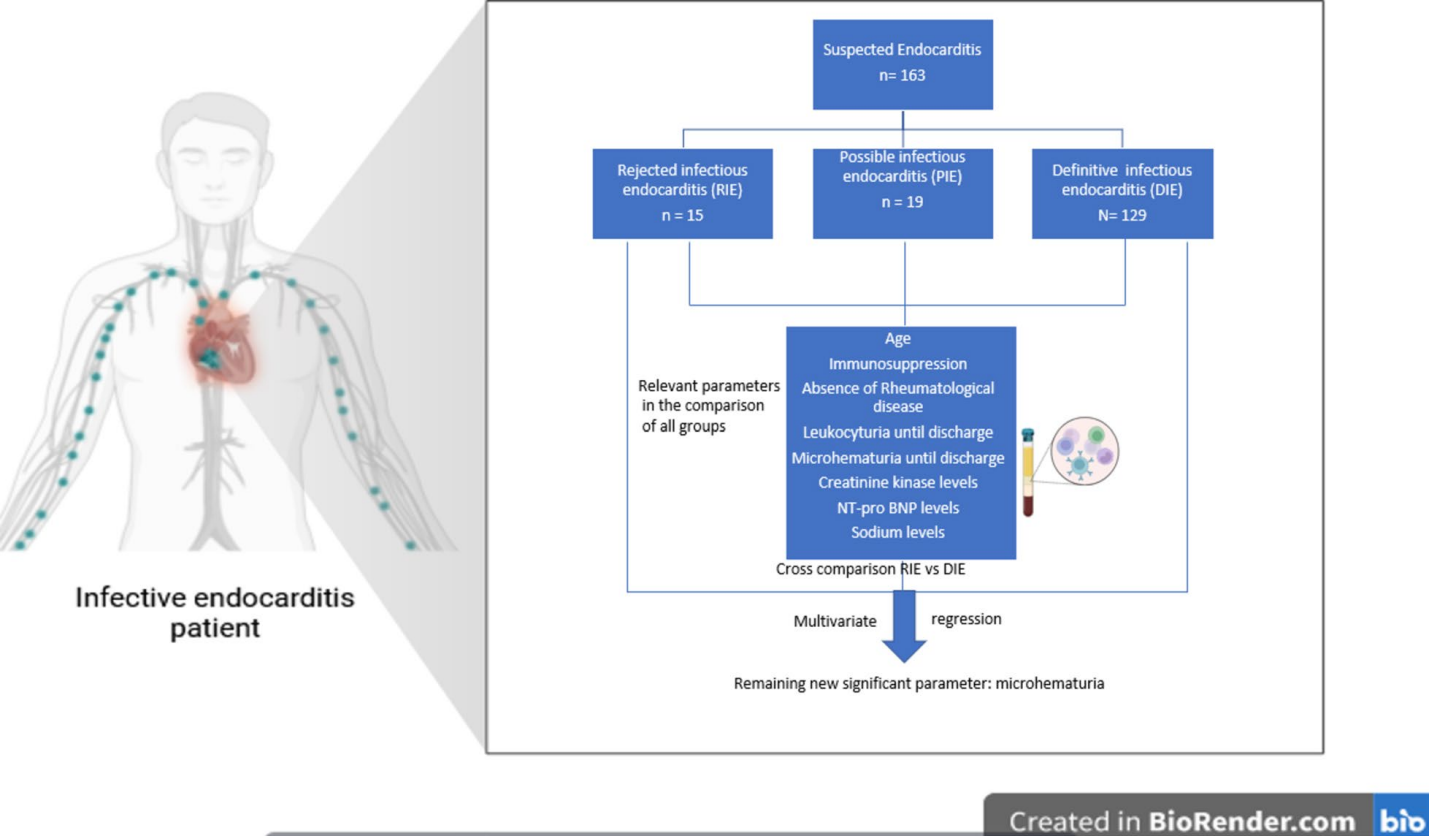
Flow chart of our analysis (created with Biorender).

The multivariate regression therefore classifies “Microhematuria” before many of the existing Duke Criteria. Assuming “Microhematuria” as a sixth minor criterion and unchanged interpretation of the Duke Criteria score, some of the patients are reclassified. As already mentioned before and as Table 7 highlights, the sensitivity and specificity of the Duke Criteria at admission were 56.1 and 40.0% in the DIE and PIE groups together. At discharge, sensitivity in the DIE and PIE groups together increased to 94.6% and specificity decreased to 33.3%. When adding microhematuria as a sixth criterion, sensitivity at admission increases to 60.1% with no changes in specificity. At discharge, sensitivity increases to 95.9% but specificity decreases by 6.6 percentage points to 26.7%. In Table 7 DIE and PIE and RIE refer to the definite diagnosis, the results with microhematuria as a minor criterion are highlighted in italic. Figure 2 summarizes the whole study.

**Table 7:** Simplified Table of Sensitivity and Specificity of the Duke Criteria at admission and discharge with and without adding microhematuria as a minor criterion in all groups.

	Admission					Discharge				
	Duke positive		Duke negative		Total	Duke positive		Duke negative		Total
DIE & PIE	83	*89*	65	*59*	148	140	*142*	8	*6*	148
RIE	9	*9*	6	*6*	15	10	*11*	5	*4*	15
Total	92	*98*	71	*65*	**163**	150	*153*	13	*10*	**163**
Sensitivity	56.1%		*60.1%*			94.6%		*95.9*%		
Specificity	40.0%		*40.0%*			33.3%		*26.7*%		

Significant values are in bold and italic.

## Discussion

In our retrospective study, we tried to analyze the timeliness of the Duke Criteria^7^ and identify new valid parameters.

The Duke Criteria represent a milestone in the diagnosis of IE as they rose validity from 49% (old Israel Beth Criteria) to 82%^14^. The intricate and heterogenous nature of current IE, coupled with the multimorbidity often observed in an aging population, necessitates a thorough evaluation of the Criteria. IE can present with a complex combination of symptoms which often leads to a prolonged time until diagnosis, which results in a delay in therapy and worse outcome^11^. Therefore this study aims to consider a potential revision or expansion of the Duke Criteria to increase their sensitivity, especially in the first days after symptom onset.

Our DIE group was representative for a cohort of patients with IE. In fact, our distribution in NVE, PVE and lead IE did not differ widly from the average in European Registers with (56.6% and 30.1% vs. 65.1% and 25.6%). Also, within the NVE, the affected valves were representative for a European IE collective.

We observed a non-significantly higher mortality in the RIE group in comparison to the DIE group (26.7% vs. 17.1% respectively). The causes of death in the RIE group mostly have been severe systemic infection and inflammation reaction with organ failure. It shows that patients with rejected IE are not necessarily healthy. Symptoms of sepsis can be similar to those of IE while having a high mortality rate and requiring a therapy initiation as fast as possible. So, if physicians err in their suspicion of endocarditis, the treatment of a potentially life-threatening condition can be simultaneously delayed. Therefore, the higher mortality in the RIE group can be explained due to potential misdiagnosis and delay of the right treatment. The mere suspicion of a patient to have an endocarditis therefore features dangerous differential diagnosis that justify caution. Patients should not be sent home without finding the cause of their symptoms.

Especially because mortality in the RIE group that does not have an endocarditis is so high, it is important to be able to rule out an endocarditis faster as the mean time until IE rejection was 8 days. From this aspect microhematuria that can lower specificity at certain times must not be helpful. With sepsis being a differential diagnosis, where an early onset of the therapy can be vitally essential, a reasonable extension of the Duke Criteria is always helpful for RIE patients as a dictum in endocarditis is to wait until microbiologic cultivation becomes positive and therefore a contradiction to general sepsis treatment.

In our analysis, the major criteria qualify as such because of their high validity. Sensitivity of the imaging criterion improves over time which supports the recommendation of repeated imaging or imaging modality change if endocarditis is still suspected in a patient where primary imaging at admission is negative^15^. The minor criteria show less validity, only vascular phenomena, which can be regarded as a complication of IE, became significant over time. Immunologic phenomena did not show a significant difference between the groups, but are neglected often. Lately, van der Vaart et al. studied the impact of immunological phenomena and could reclassify 13% of patients with suspected IE to definite IE through systematic testing according to the Duke Criteria^5^.

Regarding microbiology, there could be a reevaluation of having a minor microbiologic criterion^16^ because of a changing spectrum, the changes of the guidelines for antibiotic prophylaxis and the latest breakthroughs in lab medicine. The latest guidelines already recommend using advanced lab methods as 16S and 18S rRNA sequencing and next-generation sequencing in cases of blood-culture negative IE respectively highly suspected IE without microbiological findings^16^.

Since our design is based on a physician’s clinical assessment to suspect an endocarditis, we also could not confirm the significance and importance of predisposition and fever. At presentation in a clinic, these minor criteria are the ones that can be assessed immediately and lead to the first suspicion of IE. Every patient with a prosthetic heart valve or a central feeding line that contracts fever will be suspected to have an endocarditis. Therefore, these patients are overrepresented in our cohort and often end up with another diagnosis as they are significantly older that their peers with possible IE. We still believe that predisposition and fever are important factors in the diagnosis of IE. Because of their age, patients with predisposition have more comorbidities like heart failure or a tendency for a more frequent occurrence of vascular phenomena (*p* = 0.093), that can also be a sign of a rheumatic disease.

Looking for new potential parameter to expand the Duke Criteria, younger age, rheumatoid disease, leukocyturia and microhematuria qualified in the screening as such. We did not include age in the following analysis because of a great interquartile range overlap and because endocarditis is a disease that affects all age groups.

Rheumatoid disease was a (non-significant) protective parameter in our analysis. It shares common symptoms with IE since it is often associated with a history of unclear and unspecific fever with immunologic phenomena and seldom with degenerations of heart valves like Libman Sacks Endocarditis^17^, that can be mistaken for vegetations. Therefore, having a rheumatic disease makes an IE less probable.

In our analysis, microhematuria ends up as a potential new criterion. Advantages of urine assessment would be the non-invasive and reasonable test method with fast results and therefore a wide availability also in the outpatient sector. Nevertheless, microhematuria has a low specificity since it can be symptom of many pathologies^18^. Furthermore, in long-term hospitalized patients more samples turn unspecific due to false positive results because of catheterization or urinary tract infection. Therefore, it should be analyzed at an early stage if available.

The low specificity is also the reason why it has been rejected by the ESC as a Duke Criterion so far^7^.

Expansions of IE criteria have therefore been discussed since the establishing of the Duke Criteria. For example, the aforementioned British study of Lamas et al. tried to pave the way for such a modification of the Duke Criteria. In this study of 1997, the newly proposed parameter were “the presence of newly diagnosed clubbing, splenomegaly, splinter hemorrhages, and petechiae; a high erythrocyte sedimentation rate; a high C-reactive protein level; and the presence of central nonfeeding lines, peripheral lines, and microscopic hematuria”^4^. Because of our retrospective design, we were not able to analyze some of the aforementioned parameters but given our results we could highlight the importance of an early urine assessment.

It should be up for discussion if and how to integrate microhematuria the best way possible in the Duke Criteria. An adjusted lab test could make it an own criterion, or it could become part of the vascular or immunologic phenomena as already suggested in the 90s by Lamas et al.^4^. Van der Vaart et al. classified microhematuria as immunological phenomenon, also showing a higher incidence of microhematuria in IE patients as in RIE patients^5^.

We have to acknowledge the limitations of our study. This is a unicentric retrospective study with 163 patients that have been collected over the period of 10 years. In addition, the size of the compared groups was not the same. We tried to abide by the ESC recommendations that includes a “PIE” group. Furthermore, the classification in DIE, PIE and RIE group is based on the clinical diagnosis and is not pathologically proven in all cases. Even though this is clinical reality, it limits our study. Albeit the value of the descriptive statistic helps understand the changes that IE has undergone lately, the reach or the comparative study must still be undergirded with more analysis.

## Conclusion

Even though IE has been known for a very long time, its correct and timely diagnosis remain difficult. The Duke Criteria that are in use for around 30 years have been a useful tool for that purpose. In essence, our collective confirmed that even with their flaws, the Duke Criteria aged well. In the face of everlasting improvements in medicine, with complex valve treatments, changing bacterial and fungal spectrum, antibiotic resistance and complex differential diagnosis, they are in constant need of revision and should adapt to state of the art lab and microbiological methods and the pace of clinical practice.

## Data Availability

Since IE is a rare disease, as a matter of patient confidentiality and respect for their privacy, we have opted not to disclose any raw data so that it cannot be traced back to a patient with infectious endocarditis in this publication. However, for the sake of scientific inquiry and transparency, the data is available upon request. Any other non-personal data generated is available in this publication and its tables. Please feel free to reach out for further information. The information can be accessed upon reasonable request by contacting Dr. Sascha d’Almeida (Ulm, Germany), Sascha.almeida@uniklinik-ulm.de.

## References

[1] Taylor, J. The 2009 ESC Guidelines for management of infective endocarditis reviewed. *Eur. Heart J.* **30**(19), 2185–2186. 10.1093/eurheartj/ehp369 (2009). 19797330 10.1093/eurheartj/ehp369

[2] Keller, K., Hobohm, L., Munzel, T. & Ostad, M. A. Incidence of infective endocarditis before and after the guideline modification regarding a more restrictive use of prophylactic antibiotics therapy in the USA and Europe. *Minerva Cardioangiol.* **67**, 200–206 (2019). 30724268 10.23736/S0026-4725.19.04870-9

[3] Jensen, A. D. *et al.* Temporal changes in the incidence of infective endocarditis in Denmark 1997–2017: A nationwide study. *Int. J. Cardiol.* **326**, 145–152 (2021). 33069786 10.1016/j.ijcard.2020.10.029

[4] Lamas, C. C. & Eykyn, S. J. Suggested modifications to the Duke criteria for the clinical diagnosis of native valve and prosthetic valve endocarditis: Analysis of 118 pathologically proven cases. *Clin. Infect. Dis.* **25**, 713–719 (1997). 9314466 10.1086/513765

[5] van der Vaart, T. W. *et al.* Value of diagnosing immunological phenomena in patients with suspected endocarditis. *Infection* **51**, 705–713 (2022). 36355270 10.1007/s15010-022-01954-0 PMC10205820

[6] Cahill, T. J. & Prendergast, B. D. Infective endocarditis. *Lancet* **387**(10021), 882–893. 10.1016/S0140-6736(15)00067-7 (2016). 26341945 10.1016/S0140-6736(15)00067-7

[7] Habib, G. *et al.* 2015 ESC Guidelines for the management of infective endocarditis: The Task Force for the Management of Infective Endocarditis of the European Society of Cardiology (ESC). Endorsed by: European Association for Cardio-Thoracic Surgery (EACTS), the European Association of Nuclear Medicine (EANM). *Eur. Heart J.* **36**, 3075–3128 (2015). 26320109 10.1093/eurheartj/ehv319

[8] Habib, G. *et al.* Clinical presentation, aetiology and outcome of infective endocarditis. Results of the ESC-EORP EURO-ENDO (European infective endocarditis) registry: A prospective cohort study. *Eur. Heart J.* **40**, 3222–3232 (2019). 31504413 10.1093/eurheartj/ehz620

[9] Keller, K. *et al.* Temporal trends in the prevalence of infective endocarditis in Germany between 2005 and 2014. *Am. J. Cardiol.* **119**, 317–322 (2017). 27816113 10.1016/j.amjcard.2016.09.035

[10] Durack, D. T., Lukes, A. S. & Bright, D. K. New criteria for diagnosis of infective endocarditis: Utilization of specific echocardiographic findings. Duke Endocarditis Service. *Am. J. Med.* **96**, 200–209 (1994). 8154507 10.1016/0002-9343(94)90143-0

[11] Cahill, T. J. *et al.* Challenges in infective endocarditis. *J. Am. Coll. Cardiol.* **69**, 325–344 (2017). 28104075 10.1016/j.jacc.2016.10.066

[12] Mahabadi, A. A. *et al.* Diagnostic value of the modified Duke criteria in suspected infective endocarditis—The PRO-ENDOCARDITIS study. *Int. J. Infect. Dis.* **104**, 556–561 (2021). 33508475 10.1016/j.ijid.2021.01.046

[13] Rajani, R. & Klein, J. L. Infective endocarditis: A contemporary update. *Clin. Med.* **20**, 31–35 (2020). 10.7861/clinmed.cme.20.1.1 PMC6964163 31941729

[14] Hoen, B. *et al.* Evaluation of the Duke criteria versus the Beth Israel criteria for the diagnosis of infective endocarditis. *Clin. Infect. Dis.* **21**, 905–909 (1995). 8645838 10.1093/clinids/21.4.905

[15] Delgado, V. *et al.* A: 2023 ESC Guidelines for the management of endocarditis. *Eur. Heart J.* **44**, 3948–4042 (2023). 37622656 10.1093/eurheartj/ehad193

[16] Fowler, V. G., Durack, D. T., Selton-Suty, C. *et al.* The 2023 Duke-International Society for Cardiovascular Infectious Diseases criteria for infective endocarditis: Updating the modified Duke Criteria. *Clin. Infect. Dis.* **77**(4):518–526. 10.1093/cid/ciad271 (2023); [published correction appears in *Clin. Infect. Dis.* 2023]. 10.1093/cid/ciad271 PMC10681650 37138445

[17] Taniyasu, N., Koh, Y., Hiramatsu, T., Yokoyama, S. & Kunii, Y. Aortic valve replacement due to Libman-Sacks endocarditis combined with infectious endocarditis. *Nihon Kyobu Geka Gakkai Zasshi.* **44**(8), 1193–1197 (1996). 8828383

[18] Barocas, D. A. *et al.* Microhematuria: AUA/SUFU guideline. *J. Urol.* **204**, 778–786 (2020). 32698717 10.1097/JU.0000000000001297

